# The Impact of a Training Program Based on Next-Generation Science Standards on Scientific Inquiry

**DOI:** 10.3390/ejihpe13070087

**Published:** 2023-06-25

**Authors:** Eman Najjar, Wajeeh Daher

**Affiliations:** 1 Faculty of Educational Sciences, An-Najah National University, Nablus P400, Palestine; 2 Department of Mathematics, Al-Qasemi Academic College of Education, Baqa-El-Gharbia 3010000, Israel

**Keywords:** training program, next-generation science standards (NGSS), scientific inquiry (SI)

## Abstract

The Next-Generation Science Standards (NGSS) are standards concerned with K-12 grades. This study aimed to identify the impact of a training program based on Next-Generation Scientific Inquiry (NGSI) through training 30 science teachers and investigating the training impact on teachers’ use of inquiry in their teaching. The study attempted to answer the following question: What is the impact of a training program based on the NGSS on the inquiry practices of primary school science teachers? The study’s data collection tools included two focus groups and nine semi-structured interviews. Data analysis utilized the thematic analysis framework. The research results showed an impact of the training program on the inquiry practices of teachers via changes in the teachers’ roles: the teacher became a facilitator and motivator through planning her/his activities efficiently. Students in the training program’s science classroom developed into researchers and scientists who autonomously acquired their knowledge.

## 1. Introduction

Understanding science is substantial for school students [1]. Students need to know how to approach science learning, especially with technology [2,3,4,5,6]. Therefore, all students, from elementary to high school, must have some degree of scientific understanding to effectively participate in the technological era in which they live [7]. Studies highlight that current science learning outcomes are insufficient to adequately prepare students for life, showing a significant gap between the skills students acquire and the skills they need. The blame lies with the fact that the science curriculum objectives have become insufficient to prepare students for the current era. Responsibility rests with policy and educational decision makers in developing the science curriculum [8,9,10,11]. This means that public policy needs to take into account the different conditions of modern life, which can be conducted through sustainable education.

Eid [11] says that the objectives of school curricula varied throughout their long history. Due to the status of science and its role in advancing nations, most countries update their curricula as part of their various development programs. Thus, the science curriculum is one of the most significant curricula concerned with developing and modernizing its teaching and learning strategies. Looking at the history of science curricula, we notice that in the 1960s and 1970s, the emphasis was on teaching students science and knowledge, but gradually the focus shifted to connecting science curricula with societal needs and era challenges [12]. Accordingly, the development of science curricula has become an imperative imposed by the needs of the contemporary technological era [11]. Therefore, many developed countries have undertaken various reform projects concerning science curricula to align them with modern trends and developments.

Starting with Project 2061 (Science for All Americans) and ending with the National Research Council (NRC), a general framework for teaching science from kindergarten to twelfth grade was presented based on three pillars: disciplinary core ideas, crosscutting concepts, and science and engineering practices. The aim of this framework is to provide students with 21st-century skills, where these foundations have been integrated into what is known as the NGSS standards [13]. These standards are considered the most recent in science education in the world and they provide students with science education that is rich in scientific content, strategies, and practice [14]. Abdul Karim [15] confirms that these standards are developed based on research, which shows the inadequacy of the current science standards in keeping up with the requirements of the current era. While Eid [11] indicates that the Next-Generation Science Standards (NGSS) reflect a new vision for science education; initiatives to reform science education have started based on these standards because they are enriching, coherent, and inclusive [11].

The NGSS standards aim to revolutionize science teaching strategies to meet lifelong learning skills, such as scientific inquiry (SI) and thinking skills, which help prepare students for university and later for the labor market [9]. In this regard, Eid [11] and Abdul Karim [15] emphasize the necessity of reevaluating the professional development training programs for teachers to comprehend and implement NGSS. This will support the teacher in adopting these standards and thus make the necessary change in the student’s scientific knowledge through utilizing the SI skills. 

### 1.1. Literature Review

#### 1.1.1. Next-Generation Science Standards (NGSS)

Today, the world is witnessing rapid progress in information technology, economic competition, professional divergence, together with social, environmental, and cultural diversity. This led to recent global concern about the need for all students to achieve quality science education by focusing more than ever on deep learning and lifelong learning. NRC [16] states that the changes experienced at the beginning of the twenty-first century were taken into account when identifying the skills with which the students were required to engage in society. When the emphasis was on developing science learners in the 1960s and 1970s of the twentieth century, interest in science education emerged. Later, this concern changed to preparing students to be technologically and scientifically informed by tying social issues and needs, such as energy issues, including their sources, into science curricula. 

The previous argument indicates that science education develops learners’ use of science and technology in various fields, which helps them adapt effectively to changes in the surrounding environment [17]. The educational sector has witnessed a series of successful projects and programs to reform science education, and there have been numerous development programs over the past two decades. The most recent international standards for science education, known as the (NGSS), were developed for today’s students and the future workforce, where they focus on the interconnectedness of science and real-world contexts. Providing science education to all students at an appropriate educational level and realizing a coherent vision of science and engineering education must be enriched, focusing on deep understanding and application of content across various subjects and levels of study from kindergarten to the end of secondary school [18]. In addition to this, following the NGSS will enable policy makers to encourage active learning of science as part of sustainable education. 

#### 1.1.2. NGSS Dimensions

Scientific and Engineering Practices: Scientific practices are those that scientists use in building models and theories about the natural world, while engineering practices are used in building and designing systems [8]. Students can better understand how scientific knowledge is developed by engaging in scientific practices, while engineers can better understand their work and methods by engaging in engineering practices [19]. The NRC emphasizes that conducting scientific inquiry necessitates skills and knowledge about these capabilities by using the term “practice” rather than the similar term “skill”. The practice’s primary goal is to help students comprehend inquiry methods used by scientists and engineers rather than just the content itself [20]. According to Krajcik and Merritt [21] and NRC [22], the scientific and engineering practices are eight: (1) asking questions and identifying problems; (2) developing and using models; (3) planning and carrying out investigations; (4) analyzing and interpreting data; (5) engaging in arguments; (6) building explanations and designing solutions; (7) obtaining, evaluating, and communicating information; and (8) using mathematics, computational thinking, and computers.

Disciplinary Core Ideas (DCI): The primary role of teaching science is not to teach all the facts; it is to provide students with sufficient basic knowledge to enable them to discover knowledge on their own. NGSS focuses on a specific set of ideas central to scientific branches, which include clarifications of scientific phenomena. By focusing on the main ideas, students learn the links between concepts and principles so they can apply their understanding to future situations that may confront them, forming what is known as an “integrated understanding”; thus, supporting students in engaging in an integrated comprehension is essential; it allows students to tackle real-world problems, which adds motivation to their learning. This dimension is separated into the following primary branches: life sciences, physical sciences, earth and space sciences, engineering, and technology [17]. Omar [23] indicates that the NGSS science standards document includes 44 main ideas: 12 main ideas in physical sciences, 14 main ideas in life sciences, 12 main ideas in space sciences, and 6 main ideas in engineering sciences, technology, and applications of science.

Crosscutting Concepts (CCC): These are concepts that express science-related issues and give an organizational chart that serves as a foundation for connecting areas, revealing connections between various scientific terms, and coherently exhibiting them based on scientific foundations. They also help link different fields of science and help students discover the relationship between the four fields of science: physical sciences, life sciences, earth and space sciences, and engineering design [24]. Dusch and Bybee [25] summarize the components of CCC as follows:

Patterns: patterns are observable in nature and are subject to identification as a result of attempting to answer questions about relationships in scientific phenomena;

Cause and Effect: understanding the mechanisms and explanations through which the scientific activity takes place;

Scale, Proportion, and Quantity: understanding different sizes, proportions, energy rates, and related relationships between quantities and their change;

Systems and System Models: determining the dimensions of a system and the making of a model for developing understanding in science and engineering;

Energy and Matter: understanding the behavior of a system by tracing the flow, circulation, and conservation of energy and matter in that system;

Structure and Function: recognizing the way things are composed helps in understanding the properties and functions associated with them;

Stability and Change: understanding what causes stable natural and artificial systems and what governs how quickly they change.

#### 1.1.3. Scientific Inquiry (SI)

This is one of the most effective teaching methods for developing scientific thinking among students as it allows them to practice learning methods, processes, and investigation skills [26].

The National Association of Science Educators defines SI as activities through which students develop their knowledge and understanding of scientific ideas, as well as witness how scientists study the natural world [27]. Abdul Karim [15] defines inquiry-based science learning as a scientific teaching and learning strategy that stems primarily from understanding how students succeed in learning. It is founded on the belief that students must fully comprehend what they are learning rather than just simply repeat the information learned. Inquiry activities, as defined by Hwang et al. [28], are those in which students are encouraged to build scientific knowledge and understanding through an interactive process based on building, critiquing, and refining the experiences they gain. Students enjoy scientific activities while improving their ability to establish assumptions, make observations, and interpret data based on these observations.

#### 1.1.4. The Importance of Inquiry Activities

Educational activities have become part of modern school philosophy, which shows researchers’ interest in educational activities as leading to students’ knowledge and achievement, as well as their personalities. This interest helps reveal their preparation in the school’s various fields, and thus direct their tendencies and capabilities. According to this view, educational activities are a critical part of the curriculum in the broad sense. These activities help achieve comprehensive, integrated growth and balanced education in a way that helps students make their way in practical life. In this practical life, they rely on themselves to face difficulties and facilitate their individual and social responsibilities in achieving their society’s goals [29].

#### 1.1.5. Science Teacher Role in SI

The teacher plays a vital role in the inquiry process, which takes place in the classroom. In this process, the teacher takes care of organizing work in small groups, clarifying misconceptions, developing students’ understanding of scientific content, and linking their previous experiences with the new knowledge. Specifically, the teacher’s role in the inquiry activities is as follows: the teacher raises a problem and the students conjecture scientific hypotheses then verify the validity of the assumptions through collecting, analyzing, and interpreting information [27]. Therefore, it is important to try to strengthen the science teacher’s role in SI, together with studying the process of this strengthening, especially in the context of professional development. 

#### 1.1.6. Obstacles to Implementing SI

The biggest challenge SI faces in the learning process is the teachers’ unwillingness to adapt their teaching tactics or the educational resources employed in the learning process [30]. Brown [31] indicates that science teachers lack confidence in the educational feasibility behind SI activities because these activities may not achieve their goals. In addition, teachers feel that they are unable to control the classroom environment. Fast and Jans [32] stress that science teachers need more time to improve their ability to adapt to investigative activities. Time is one of the main problems that prevent the practical application of the SI because of the short period for the lesson, which makes teachers unable to give students enough time to answer the questions. Thus, the teachers resort to using several simple questions that do not require the employment of higher-order thinking skills [33].

### 1.2. Research Goals, Rationale, and Questions

Palestinian schools attempt to keep up with scientific and technological progress in the science teaching field. NGSS is one of the most recent reform movements in teaching science, transforming the classroom from a place where students learn science to one where they produce science [34]. For a science teacher to be able to implement these standards, he or she needs new strategies for designing SI activities and teaching them. Because deep comprehension is the foremost step in developing high-quality curricula, teachers must have complete support to comprehend these standards [7]. 

The research problem stems from the results of international science tests, named the national tests, which measure students’ life skills through questions that focus on SI skills. The students’ achievement has been unsatisfactory. In addition, low outputs of national science tests, which measure the cognitive skills of students, persist. Furthermore, the science education literature in the Palestinian schools confirm the existence of a gap between expected outputs and inputs. Here, the authors realize that teaching science needs to address a qualitative shift, which will help develop, improve, and reform science teaching throughout professional development programs [35]. Thus, there is a need for a NGSS-based training program in the Palestinian context, together with accompanying research that studies the changes that such a program enables. 

This study comes in response to international recommendations from previous studies [7,8,9,15] that call for studying the NGSS and its role in preparing learners for conceptual learning in science. The study will contribute to shedding light on NGSS in the Palestinian context, especially since current science standards are too weak to keep up with the challenges of the current era or build a training program based on these standards. The current study is a response to the need for a training program based on the NGSS to help science teachers understand these standards and comprehend their implementation mechanisms in the classroom.

The indicators of international studies’ findings suggest that teaching science in Palestinian schools has a serious problem [35]. These findings must be taken into account when developing science education. In this regard, the most recent programs for science reform and development have acknowledged the need for a genuine and all-encompassing approach, which can be accomplished by utilizing NGSS. In the frame of this research, we aim to develop a training program based on the NGSS and determine the changes promoted by this training program in SI practices among science teachers.

Studies have confirmed the importance of the science teacher’s involvement in training programs in general [36,37] and NGSS in particular. Morales [38] argues that teachers need to understand NGSS and its application in the classroom. In this study, we aim to answer the following overarching research question:

What changes does a training program based on the NGSS promote in the SI practices of science teachers in Palestinian schools?

## 2. Materials and Methods

### 2.1. Research Context and Participants

Qualitative research involves collecting, analyzing, and interpreting data that are not easily reduced to numbers [39]. This research utilizes constant comparison between the units of data to suggest codes relevant for the topic of the research, puts similar codes into one category, and then puts similar categories into a theme. In the present research, semi-structured interviews and focus groups are the tools used to gather data from the participants in the training program about their use of SI in teaching science, with a simple random sample of 30 science teachers for grades 6–8.

#### The NGSS-Based Training Program

The training program was designed taking into account the educational literature for NGSS, represented in two key documents: the framework of K-12 science education and the science standards book for the next generation. The focus in these two documents was integrating scientific and engineering practices and the application of popular concepts into science from kindergarten to twelfth grade.

The proposed training program was designed considering the NGSS and the ADDIE model; accordingly, the training program content and objectives were determined in addition to the expected training outcomes for science teachers when implementing the program. The following educational content, sub-objectives, and training outcomes were included in the NGSS-based training program: tracing the history of the NGSS; developing an NGSS procedural definition; concluding the goal of implementing NGSS in teaching science; interpreting the vision of teaching science considering NGSS; identifying the disciplinary core ideas of a subject from the school curriculum; defining the CCC of a specific topic; and defining the CCC of a relevant topic.

A group of experts provided feedback on the training program, and the program was developed in accordance with the feedback. The first author implemented the training program in early August 2022 and continued through the end of October 2022. The program consisted of 30 training hours divided into face-to-face and online meetings, in addition to the carrying out of a set of tasks during each training meeting.

The following is an SI-based activity that the participants worked with and discussed during the educational program.


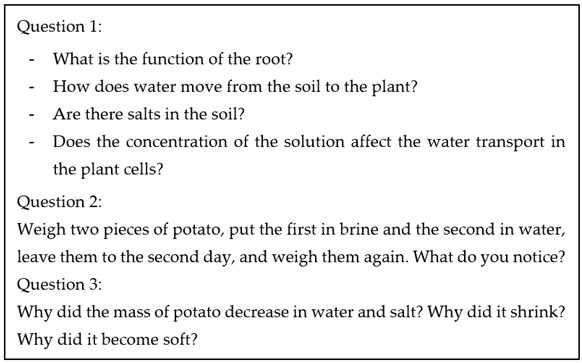


### 2.2. Data Collection Tools

#### 2.2.1. Semi-Structured Interview

The interview aims to collect detailed investigations of science educators’ perspectives and understanding of SI and the changes it promotes in teachers’ practices in the classroom. An interview is an appropriate tool for this research because it allows the researcher to explore participants’ descriptions and plans and generate unexpected insights into teachers’ opinions [40,41]. The interview was conducted with six randomly selected teachers at the end of the training program with open-ended questions (see Table 1 for the description of these teachers). 

The interview questions were initially general and related to science education in general; thus, they could easily create a context for their beliefs about SI [41]; then, the interview proceeded to in-depth, open-ended questions concerning teachers’ understanding of the practice of scientific inquiry in the classroom, the instructions during NGSS implementation, and the challenges they might face during implementation. Thus, detailed information regarding the perspective and understanding of the SI was collected. Each participant was interviewed once. Each interview lasted from (50–60) minutes. These interviews gave the teachers time to answer questions more specifically about their experience with SI and provided qualitative data vital to the research. The interviews were held via Zoom. The meeting’s goal was clarified in the beginning by going over the interview protocol with the participants.

#### 2.2.2. Focus Group

The present study used focus groups to obtain qualitative data through the participants’ interaction. Two focus groups were conducted, each with six teachers who were chosen at random and did not engage in individual interviews (see Table 2 for the description of these teachers).

The focus groups were conducted at the end of the training program, and the questions were open-ended. These focus groups provided the teachers with time to respond more specifically about their experience with the SI. The two focus groups were held via Zoom, with each interview lasting 90 min. In the beginning of each focus group’s meeting, the purpose of the meeting was clarified by the coordinator.

### 2.3. Data Analyses

Thematic analysis of data collected through individual interviews and focus groups focuses on both identifying a pattern through inductive reasoning by repeatedly examining and comparing the collected data to reduce it to a set of topics or categories and then generating, reviewing, defining, and interpreting these categories [39]. Thematic analysis is appropriate here because there is no previous research on the application of NGSS in the West Bank. The objective analysis throughout the thematic process is time-consuming as it requires an in-depth reading of the data [42]. To illustrate the analysis process, let us give an example on the sub-theme “Increasing students’ role in learning”. At the beginning, we noticed participants’ saying things such as “my students increased their role in learning”, “the students decided the solution method”, or “the SI activities made my students more involved in their learning”. We gathered these statements together as they all indicate the same idea, i.e., that of increasing the students’ role, so we gathered these statements under the sub-theme “Increasing students’ role in learning”. We performed the same constant comparison of the statements to arrive at the sub-theme: “Increasing teachers’ content coverage”. In addition, we noticed that both the sub-themes resulted from the change of the teacher’s role into that of guidance and facilitation. So, we put the two sub-themes under the theme “guidance and facilitation”. 

### 2.4. Trustworthiness

Trustworthiness is one way in which researchers can persuade themselves and readers that their research findings are worthy of attention. It consists of different components, such as the credibility, transferability, dependability, and confirmability of the research findings [41]. To enhance credibility, interviews and focus groups have been used for data triangulation, and participants were given a transcript of the interview and allowed to clear up any perceived misinterpretations; this also allowed the participants to volunteer additional information that may have been relevant to the study. Transferability was achieved by providing sufficient information in the form of a “thick and clear description” that would allow readers to interpret the phenomenon under study. To enhance dependability, we provided adequate contextual information about each participant, which would make the study replicable by other researchers. In addition, the interview questions are detailed, whether for the interview or for the focus group. Moreover, details of the data collection and data analysis are given. The findings are not biased but accurately portray the participants’ responses.

## 3. Results

The results of six individual interviews and two focus groups were analyzed using thematic analysis to answer the research question. Table 3 shows the educational themes and sub-themes that emerged as characterizing teachers’ practice of NGSS activities.

Below, we describe each theme and sub-theme and add appropriate quotations from the participants.

### 3.1. Guidance and Facilitation

Teachers’ guidance of their students’ learning consists of two sub-themes: increasing students’ role in learning and increasing teachers’ content coverage.

#### 3.1.1. Increasing Students’ Role in Learning

Halima mentioned in the interview how teachers’ roles changed to be more aligned with guidance and facilitation of students’ work towards student-centered learning: “In SI activities, I present the concept to the students in a real-life problem context; then the students begin to discuss and ask questions and propose hypotheses and solutions; students begin to test these hypotheses until they reach a solution as a result of searching for scientific explanations. Specifically, the percentage of student-centered activities in lectures increased”. Thus, Halima’s statement shows how the real-life context that is part of the NGSS activities contributes to the change in students’ roles, wherein the students became more active in their learning.

#### 3.1.2. Increasing Teachers’ Content Coverage 

Amal mentioned in the interview how the program supported her coverage of the content, saying: “the percentage of my implementation of activities changed after I joined the program; it became equivalent to 60% after it was 40%, it became student-centered, as my role decreased in lecturing and increased as a facilitator and supporter”. Amal’s statement shows how student-centered learning helped the teacher increase the content coverage. 

### 3.2. Stimulating Students’ Motivation and Curiosity

The category “stimulating students’ motivation and curiosity” consisted of two sub-themes: NGSS activities’ encouragement of students’ curiosity and NGSS activities’ encouragement of varied-ability students’ motivation.

#### 3.2.1. NGSS Activities’ Encouragement of Students’ Curiosity

The participating teachers talked about how NGSS activities encouraged students’ curiosity as they engaged in inquiry. Amal said in the interview that students needed something that fascinated them and kept their curiosity: “I was able to encourage students’ curiosity as the NGSS activities involved inquiry. I saw that the students were curious to follow the learning topic, especially with the technological tools”. As Amal argued, using technology as a tool for inquiry helped the teacher turn the science class into one of curiosity, which motivated students’ learning.

#### 3.2.2. NGSS Activities’ Encouragement of Varied-Ability Students’ Motivation

Teachers talked about how NGSS activities helped motivate students despite their varied abilities. Fidaa said in the interview: “I was able, through the activities of the NGSS, to involve all my pupils, despite their various skills, in carrying out the inquiry activity while they efficiently worked in groups. This is evidence that NGSS activities made them motivated to do the activity passionately”. Thus, NGSS activities enabled the teacher to support students of varied abilities in carrying out the activities as they became motivated.

### 3.3. Supporting Students’ Knowledge and Skills 

The theme “supporting students’ knowledge and skills” consists of two themes: “supporting students’ acquiring of knowledge” and “supporting students’ acquiring of skills”. 

#### 3.3.1. Supporting Students’ Acquiring of Knowledge

The participating teachers stressed that the NGSS activities provided their students with opportunities to acquire knowledge. Fida declared in the focus group: “the NGSS activities encouraged the student to discover the information himself, not just memorize it. The student worked with the tool to solve the activity, which made the scientific concepts or relations comprehendible”. The experience of Fidaa, as a science teacher, taught her that, through NGSS activities, she could support the student in acquiring scientific knowledge, specifically with the help of tools. 

#### 3.3.2. Supporting Students’ Acquiring of Skills 

The participating teachers stressed that the NGSS activities allowed them to provide their students with opportunities to acquire learning skills. Halima said in the focus group: “The NGSS activities enable me to ask the student to perform the activity himself, which gives the student the skills of self-learning, such as putting forth hypotheses and utilizing tools to verify these hypotheses”. Thus, the NGSS activities enabled the teacher to help her students acquire the knowledge and learn the skills that helped them retain this knowledge. 

### 3.4. Efficient Planning of Activities 

The theme “Efficient planning of activities” consisted of two sub-themes: “change of planning” and “following diversifying practices during the activity”.

#### 3.4.1. Change of Planning

The participating teachers reported in the interview and focused groups that their planning of activities changed as a result of their introduction to the NGSS activities. Tamer said in the focus group: “The daily planning (preparing the lesson in writing) changed from general planning to specific, clear, and effective planning; the structure of the activities made me realize what teaching strategies I need to implement and what tools the students need to implement”. Tamer argued that the framework of the NGSS activities made teachers aware that the teaching and learning strategies required the teacher’s attention while preparing classroom activities.

#### 3.4.2. Following Diversifying Practices during the Activity

Participants stated that NGSS activities enabled them to vary their teaching practices. Anwar, in the interview, compared her class before and after joining the program, saying: “In the training program, it is 60% lecture kind. It began with an introduction reminding students of what was presented in the previous class, then explained the activity, its steps, tools, and materials that would be used. Then, I carried out a scientific presentation. But after joining the training program, I start preparing for brainstorming, asking questions, and receiving questions from students, then distributing students into groups; each group explains what sources and tools they will be using, then discusses results and asks questions to reach explanations”. Thus, NGSS activities show the teachers how to diversify their teaching processes, which concerns teaching techniques in principle.

### 3.5. Scientific Debate 

After being exposed to NGSS activities, the participants reported paying closer attention to the scientific discussions of the students. Ayat stated in the focus group discussion: “I currently concentrate on the practice of scientific debate with students rather than just asking and responding to questions”. Thus, due to their increased awareness of NGSS activities, teachers started to pay closer attention to their students’ scientific discussions.

### 3.6. The Scientist Student 

The scientist–student theme consists of three sub-themes: the scientist’s practice, the student’s transferring of knowledge, and the multi-thinking student.

#### 3.6.1. The Scientist Practice

The participating teachers mentioned that the NGSS activities turned the students’ work into scientific practice. Neveen stated in the focus group: “before joining the training program if students asked a question, I used to answer them directly, but after joining the program, I encouraged them to formulate hypotheses and provide the resources they needed to test these hypotheses; I call the students young researchers; in this program, students play the role of scientists”. Thus, the NGSS-based program, as a result of the inquiry-based activities, enabled teachers to consider students as young scientists. 

#### 3.6.2. Students’ Transferring of Knowledge

In the interview, Soha emphasized: “the SI activities helped the student consolidate the information since the student created tools and materials, generated hypotheses, tested them, and got the findings. The student would then apply and transfer to practical life”. Soha believed that SI activities not only helped the student to conceive knowledge but also to consolidate this knowledge. 

#### 3.6.3. The Higher-Order-Thinking Student

The participating teachers talked in the focus group discussions about how the NGSS activities helped motivate the students’ multi-thinking. Fidaa said, “SI activity implementation developed students’ scientific thinking, encouraged their critical thinking, and helped teachers build a student who is a critical thinker, strengthening his or her ability to reach conclusions. the student who uses ideas to solve science problems activates creative ideas to arrive at ideal solutions to the problems”. Thus, the inquiry-based activities that required high-order thinking from the students encouraged them to use high-order thinking as creative and critical. 

## 4. Discussion

Science teaching is milestone for school students [43,44], so it is necessary to engage science teachers in training program to advance their teaching [45]. The present research was intended to verify the impact of a training program on science teachers’ practices. The results indicate that the participating teachers show favorable views of the training program and the changes it promotes in their SI practices. Specifically, the results show that the role of the teacher becomes that of a guide and facilitator. The teacher can be a director, supervisor, and facilitator of the student’s advancement toward achieving the goal. This interpretation is in line with the NGSS’s [16] affirmation of the importance of the teacher’s ability to adapt to changing roles. Waters [46] claims that in light of the NGSS, the teacher in the SI gives up the duty of providing all the information necessary to take on the role of facilitator by spending less time lecturing.

The results show that taking care of the diversified students is one of the modified teacher roles; these results can be supported by adhering to SI requirements, together with considering each student’s personality and learning preferences. This interpretation is consistent with what OkheeLee et al. [47] express regarding the implementation of the standards for heterogeneous groups (gifted, intelligent, students with special needs, students with slow learning, and students who are not native English speakers); they stress that all groups can engage in scientific work, with a change in performance at all levels.

Likewise, the results show that the teachers start to vary their strategies in response to the NGSS requirements and conditions; the effect can be explained by shifting the teacher’s perspective and the classroom’s practices to put more emphasis on ideas conceiving, crosscutting concepts, and scientific procedures. This interpretation is consistent with what Hwang et al. [28] say about SI, as one of these techniques focuses on researching, questioning, watching videos, and adopting inquiries. According to Al-Adima [48], NGSS provides a quantum leap in the teacher’s practice, allowing students to shift from a position where they learn science to a place where they work in science explorations. Furthermore, the result shows that the teacher diversifies the strategies; this result can be explained by the teacher’s thinking and classroom practice shifting from traditional education to education that focuses on the core ideas of integration, crosscutting concepts, and scientific strategies.

The findings demonstrate how skillfully and efficiently the teacher plans the activities. The Qablan study [49] confirms teachers’ abilities to prepare plans and design lessons oriented toward inquiry and apply them in the classroom as a result of their enrollment in a professional development program, which supports the interpretation that teachers benefited from their program participation by planning lessons oriented toward SI application.

The results show that teachers can develop their skills in SI as they use it among their students and find it beneficial for them. These results can be explained by the SI approach in light of the NGSS, which emphasizes the necessity of the science teacher’s inquiry abilities. This interpretation is in line with the emphasis Al-Adima [45] places on the five-year learning model’s (5Es) contribution to the development of SI skills through the measurement, control, and elaboration of hypotheses. The 5Es model was used in the NGSS training program, which focuses on achieving SI skills through the productive application of the eight science practices [50].

In addition to the above, the results suggest that students end up becoming scientists. This outcome can be explained by the processes in which students create and test hypotheses and recognize that they are genuine scientists or have the potential to become scientists for the rest of their lives. This interpretation is consistent with the definition of SI by Tyler and Britton [51] and Waters [46], namely, as the process by which scientists investigate their surroundings and create hypotheses and supporting data to assess and transmit information. The student’s function shifted dramatically from a vessel for memorizing knowledge to a multi-thinking learner. 

In order to create new knowledge that can be used to nurture students’ understanding of science ideas, the NGSS aims to equip students with critical, logical, creative, and generative thinking skills. According to Richards et al. [52] and Waters [46], implementing SI activities within the context of the NGSS leads to significant growth in students’ thinking. 

Additionally, the fact that students who take part in SI activities will have more opportunities to study a variety of educational resources can be used to explain this outcome. This interpretation is in line with the NGSS’s belief that students can learn, develop into more discerning consumers of scientific information, and eventually become researchers by engaging in research [13,16,25,34].

The result reveals the role of the student as a knowledge transferor. Given that the majority of scientific issues are connected to actual natural phenomena, this result can be explained via the activity of the student who participates in solving real scientific problems that can transfer knowledge. This interpretation is in line with the NGSS’s view of the significance of the student’s participation in SI and the role it plays in developing students who can apply what they have learned and acquired in their everyday lives [29,53].

## 5. Conclusions and Recommendations

The practices of science are at the center of educational researcher [54,55,56,57], especially the practices of science teachers [58,59]. The present study intended to examine the change in science teachers’ practices as a result of participating in a professional development program that focuses on NGSS SI activities. The research results indicated that the NGSS-based program resulted in a change in the participating teachers’ practices. The SI activities made teachers’ practices move towards more student-centered practices, which made the students’ practices similar to those of the scientist. Thus, there is a need to raise the education of the science teacher towards using NGSS-based activities, which could be achieved by joining training programs according to NGSS standards. 

The present study results also showed that NGSS activities could support the student in acquiring scientific knowledge, especially with the help of tools and resources. Thus, there is a need to train science teachers to use scientific inquiry in science instruction in light of the NGSS, with an emphasis on providing tools and resources to design SI activities. The training programs need to focus on the benefits and potentialities of scientific inquiry that utilizes tools and resources. Changing teachers’ attitudes is the first step towards changing their practices, so training programs should focus on this change of teachers’ attitudes towards NGSS -based SI activities. 

In addition to the above, policy makers in ministries of education need to pay attention to the NGSS and consider NGSS-based activities when writing directions for science curriculum developers; this will enable science teaching and learning to be founded on sustainable education. 

## 6. Study Limitations

The present study was conducted among a sample of 30 science teachers for grades 6–8. There is a need to replicate the present study with different groups of science teachers, which could consolidate the results of this study. In addition, there is a need to investigate the change of teachers’ SI practices from the view of the students, which triangulate the present study findings. 

The present study addresses teachers’ practices in grades 6–8; there is a need to verify the change in teachers’ practices as a result of training programs in other school phases such as secondary and primary schools. 

The present study utilizes interviews and focus groups as sources of data. Future research needs to examine the issue of utilizing NGSS-based training program using quantitative methods as quantitative methods give significant results which could triangulate the present study’s results. 

The present study addressed the change in teachers’ practices in general. Future research is needed to address the change of specific practices among science teachers; for example, teachers’ reflections or teachers’ attitudes/beliefs regarding the use of SI activities in the science classroom. NGSS-based SI activities are expected to influence the change in other variable of teachers’ instruction. 

## 7. Ethical Considerations

The ethics issues have been widely considered throughout the research process, and participation in the training program was optional. All participants were requested to sign a consent form that included clarifications about the study’s goal and confirmation of confidentiality concerns regarding their inclusion in the study. They were free to participate as volunteers in the study and could opt out without consequences. Participants were informed of the interview and focus group procedures, which took place via the Zoom App. Consent included statements about participants’ rights to review the interview and focus group report, provide feedback, and add comments; this led to accurate and honest analysis and presentation of the collected data. The study caused no harm to the participants, and their responses will be used for research purposes. In addition, the research was approved by the IRB committee at An-Najah National University. 

## Figures and Tables

**Table 1:** Description of teachers who participated in the individual interviews.

Name	Age	Certificate	Years of Experience
Niveen	38	Master of science education	19
Amal	35	Bachelor of chemistry	7
Nida	44	Bachelor of physics	20
Lina	35	Master of science education	7
Haneen	30	Bachelor of biology	4
6	32	Master of science education	6

**Table 2:** The description of teachers who participated in the focus groups.

Name	Age	Certificate	Years of Experience
		The participating teachers in the first focus group	
Ayat	42	Bachelor of Chemistry	17
Rasha	33	Bachelor of Physics + Master of Science Education	10
Reham	35	Bachelor of Physics + Master of Science Education	12
Suha	30	Bachelor of Physics	7
Nidaa	40	Bachelor of Physics + Master of Science Education	17
Hana	41	Master of Science Education	18
The participating teachers in the second focus group			
Amal	43	Bachelor of Physics + Master of Science Education	20
Thamer	40	Master of Science Education	15
Halema	44	Bachelor of Physics + Master of Science Education	20
Fida	27	Bachelor of Science	4
Nahed	41	Master of Science Education	17
Anwar	40	Bachelor of Physics + Master of Science Education	16

**Table 3:** Emerging themes and sub-themes.

Theme	Sub-Themes
Guidance and Facilitation	Increasing students’ role in learning
	Increasing teachers’ content coverage
Stimulating students’ motivation and curiosity	NGSS activities’ encouragement of students’ curiosity
	NGSS activities’ encouragement of varied-ability students’ motivation
Supporting students’ knowledge and skills	Supporting students’ acquiring of knowledge
	Supporting students’ acquiring of skills
Efficient Planning of Activities	Change of planning
	following diversifying practices during the activity.
Scientific debate	
The Scientist Student	The scientist practice
	Students’ transferring of knowledge
	The higher-order-thinking student

## References

[1] Alshamali M.A., Daher W.M. (2016). Scientific reasoning and its relationship with problem solving: The case of upper primary science teachers. Int. J. Sci. Math. Educ..

[2] Daher W. (2009). Preservice teachers’ perceptions of applets for solving mathematical problems: Need, difficulties and functions. J. Educ. Technol. Soc..

[3] Daher W. (2010). Mathematics learning community flourishes in the cellular phone environment. Int. J. Mob. Blended Learn..

[4] Daher W., Sabbah K., Abuzant M. (2021). Affective engagement of higher education students in an online course. Emerg. Sci. J..

[5] Daher W., Omar A., Swaity H., Allan B., Dar Issa S., Amer Z., Halabi A. (2022). Upper-Basic Schoolteachers’ Beliefs about Their Students’ Awareness of Digital Citizenship. Sustainability.

[6] Daher W., Abo Mokh A., Shayeb S., Jaber R., Saqer K., Dawood I., Bsharat M., Rabbaa M. (2022). The Design of Tasks to SuitDistance Learning in Emergency Education. Sustainability.

[7] Afifi M. (2019). A proposed program based on Next Generation Science Standards (NGSS) to train middle school science teachers in the use of Science and Engineering Practices (SEPs) while teaching science. Educ. J. Coll. Educ..

[8] Al-Sadiq M., Abu Shuqair M., Alostath M. (2021). The effectiveness of a training program based on Next Generation Science Standards (NGSS) in developing scientific teaching practices among science teachers in Gaza. J. Islam. Univ. Educ. Psychol. Stud..

[9] Jad Al-Haq N. (2021). Proposed program based on next generation science standards. J. Coll. Educ. Educ. Sci..

[10] Obaid M., Suleiman S., Almosheki K. (2020). The effectiveness of a training program based on employing the educational portal in developing the skills of the administrative staff in basic education schools in Dhofar Governorate. J. Educ. Psychol. Res..

[11] Eid S. (2021). A proposed program in Earth and Space Sciences based on the Next Generation Science Standards (NGSS) to develop design thinking and some engineering habits of mind among middle school students. Educ. J. Coll. Educ..

[12] Kang E.J.S., McCarthy M.J., Donovan C. (2019). Elementary teachers’ enactment of the NGSS science and engineering practices. J. Sci. Teach. Educ..

[13] Channell A., Cobern W., Rudge D., Bentz A. (2021). Teacher and Parent Perspectives on NGSS Alignment Following Teacher Professional Development. Sci. Educ. Int..

[14] Peltzman A., Rodriguez N. (2013). Next Generation Science Standards: Adoption and Implementation Workbook.

[15] Abdul Karim S. (2017). A training program based on Next Generation Science Standards (NGSS) to develop deep understanding, scientific investigation skills, and scientific debate among elementary science teachers. J. Arab. Stud. Educ. Psychol..

[16] National Research Council (2015). Guide to Implementing the Next Generation Science Standards. https://nap.nationalacademies.org/catalog/18802/guide-to-implementing-the-next-generation-science-standards.

[17] Al-Ahmad N., Al-Bogami M. (2017). Content analysis of physics textbooks in the Kingdom of Saudi Arabia in the light of the Next Generation Science Standards NGSS. Jordanian J. Educ. Sci..

[18] Achieve (2017). Next Generation Science Standards: District Implementation Workbook.

[19] Abu Athra S. (2019). The reality of the practice of physics teachers in the secondary stage of the standards of the next generation. Umm Al-Qura Univ. J. Educ. Psychol. Sci..

[20] Izz Al-Din S. (2018). Activities based on Next Generation Science Standards (NGSS) to develop scientific and engineering practices, critical thinking, and scientific inclinations in science among primary school students in Saudi Arabia. Egypt. J. Sci. Educ..

[21] Krajcik J., Merritt J. (2012). Engaging students in scientific practices: What does constructing and revising models look like in the science classroom?. Sci. Teach..

[22] National Research Council (2012). A Framework for K-12 Science Education: Practices, Crosscutting Concepts, and Core Ideas.

[23] Omar A. (2017). Evaluating the content of life sciences curricula at the secondary level in the Arab Republic of Egypt considering the Next Generation Science Standards (NGSS). Egypt. J. Sci. Educ..

[24] Hassanein B. (2016). Science standards for the next generation. Educ. J. Fac. Educ. Sohag.

[25] Duschl R.A., Bybee R.W. (2014). Planning and carrying out investigations: An entry to learning and to teacher professional development around NGSS science and engineering practices. Int. J. STEM Educ..

[26] Moeed A. (2013). Science investigation that best supports student learning: Teachers’ understanding of science investigation. Int. J. Environ. Sci. Educ..

[27] Ødegaard M., Haug B., Mork S.M., Sørvik G.O. (2014). Challenges and support when teaching science through an integrated inquiry and literacy approach. Int. J. Sci. Educ..

[28] Hwang G.-J., Tsai C.-C., Chu H.-C., Kinshuk K., Chen C.-Y. (2012). A context-aware ubiquitous learning approach to conducting scientific inquiry activities in a science park. Australas. J. Educ. Technol..

[29] Al Waher M. (2020). New Directions in Teaching Science, Scientific and Engineering Practices.

[30] Mathison H.R. (2011). Implementing Professional Development: A Case Study of Mathematics Teachers Using Inquiry in the Classroom Context. Ph.D. Thesis.

[31] Capps D.K., Crawford B.A. (2013). Inquiry-based professional development: What does it take to support teachers in learning about inquiry and nature of science?. Int. J. Sci. Educ..

[32] Fast J., Jans M.S. (2012). How Does the Inquiry Learning Method Affect Student Cognitive Development at Varying Ages?. Master’s Thesis.

[33] Al-Khayat M.M. (2012). The Levels of Creative Thinking and Metacognitive Thinking Skills of Intermediate School in Jordan: Survey Study. Can. Soc. Sci..

[34] Whittington K.L. (2017). How does a Next Generation Science Standard Aligned, Inquiry Based, Science Unit Impact Student Achievement of Science Practices and Student Science Efficacy in an Elementary Classroom?. Ph.D. Thesis.

[35] Mullis I.V. (2012). TIMSS & PIRLS International Study Center, Boston College: Chestnut Hill, MA, USA. https://timssandpirls.bc.edu/timss2011/downloads/T11_IR_Mathematics_FullBook.pdf.

[36] Pamuk S., Elmas R., Saban Y. (2022). A Modeling Study on Science Teachers’ Sustainable Development Knowledge, Attitudes and Practices. Sustainability.

[37] Paulauskaite-Taraseviciene A., Lagzdinyte-Budnike I., Gaiziuniene L., Sukacke V., Daniuseviciute-Brazaite L. (2022). Assessing Education for Sustainable Development in Engineering Study Programs: A Case of AI Ecosystem Creation. Sustainability.

[38] Morales C.J. (2016). Adapting to National Standards: The Experience of One Middle School Science Teacher’s Implementation of the Next Generation Science Standards (NGSS). Ph.D. Thesis.

[39] Creswell J.W., Creswell J.D. (2017). Research Design: Qualitative, Quantitative, and Mixed Methods Approaches.

[40] Kvale S., Brinkmann S. (2015). Interviews: Learning the Craft of Qualitative Research Interviewing.

[41] Seidman I. (2013). Interviewing as Qualitative Research: A Guide for Researchers in Education and the Social Sciences.

[42] Saleh H.A. (2018). A Study of the Effectiveness of the Next Generation Science Standards Implementation at a Private US Curriculum School in Dubai, UAE. Ph.D. Thesis.

[43] Lucena-Anton D., Fernandez-Lopez J.C., Pacheco-Serrano A.I., Garcia-Munoz C., Moral-Munoz J.A. (2022). Virtual and Augmented Reality versus Traditional Methods for Teaching Physiotherapy: A Systematic Review. Eur. J. Investig. Health Psychol. Educ..

[44] Daher W., Hashash I. (2022). Mathematics Teachers’ Encouragement of Their Students’ Metacognitive Processes. Eur. J. Investig. Health Psychol. Educ..

[45] Diez R., Dominguez A., Ponsoda S., Ortuño B. (2021). Social Science Pedagogy as a Way of Integrating Sustainable Education and Global Citizenship into the Initial Training of Pre-Primary Teachers. Eur. J. Investig. Health Psychol. Educ..

[46] Waters T.L. (2018). The Effects of the Next Generation Science Standards (NGSS) on Teaching Practices: An Instrumental Case Study. Ph.D. Thesis.

[47] OkheeLee E.C. (2014). Miller & Rita Januszyk. Next Generation Science Standards: All Standards, All Students. J. Sci. Teach. Educ..

[48] Al-Adima S. (2020). A Proposed Training Program Based on the Next Generation Science Standards (Ngss) To Develop the Teaching Performance of Science Teachers and Its Impact on Developing Problem-Solving Skills and Future Thinking Among Middle School Students. Ph.D. Thesis.

[49] Qablan A. (2016). Teaching and learning about science practices: Insights and challenges in professional development. Teach. Dev..

[50] NGSS Lead States (2013). Next Generation Science Standards: For States, by States.

[51] Tyler B., Britton T. (2018). Developing District Plans for Ngss Implementation: Preventing Detours and Finding Express Lanes on the Journey to Implement the New Science Standards.

[52] Richards J., Johnson A., Nyeggen C.G. (2015). Inquiry-based science and the next generation science standards: A magnetic attraction. Sci. Child..

[53] Alebous T. (2021). The level of understanding scientific and engineering practices in light of the next generation science standards among preservice student teachers. Educ. Res. Rev..

[54] Alfahel E., Daher W., Anabousy A. (2023). Students’ motivation to study science: The case of Arab students in Israel. Eurasia J. Math. Sci. Technol. Educ..

[55] Daher W., Saifi A.G. (2018). Democratic practices in a constructivist science classroom. Int. J. Sci. Math. Educ..

[56] Daher W., Alfahel E., Anabousy A. (2021). Moderating the Relationship between Student’s Gender and Science Motivation. Eurasia J. Math. Sci. Technol. Educ..

[57] Wolff-Seidel S., Budke A. (2022). Self-Assessment of Students of Geography Education and Primary Social and Science Teaching towards the Use of Digital (Geo-) Media for Written and Oral Argumentation. Eur. J. Investig. Health Psychol. Educ..

[58] Rodríguez-Nogueira Ó., Leirós-Rodríguez R., Quiroga-Sánchez E., Álvarez-Álvarez M.J., Álvarez-Barrio L. (2021). Perceptions and Degree of Satisfaction with the Health Sciences University Educational Community Regarding the Measures Adopted for the Prevention of COVID-19 in the Academic Year 2020/2021. Eur. J. Investig. Health Psychol. Educ..

[59] Valdivieso J.A., Carbonero M.Á., Martín-Antón L.J., Freitas A. (2013). Perception of School Life Skills in University Education: Comparative Analysis of the Construct Validity Between Spain and Brazil. Eur. J. Investig. Health Psychol. Educ..

